# *COVID-19 Stats:* Percentage of Middle and High School Students Aged 13–21 Years Attending In-Person Classes Who Reported Observing Fellow Students Wearing a Mask All the Time,* by School Setting and Activity — United States, October 2020

**DOI:** 10.15585/mmwr.mm7006a5

**Published:** 2021-02-12

**Figure:**
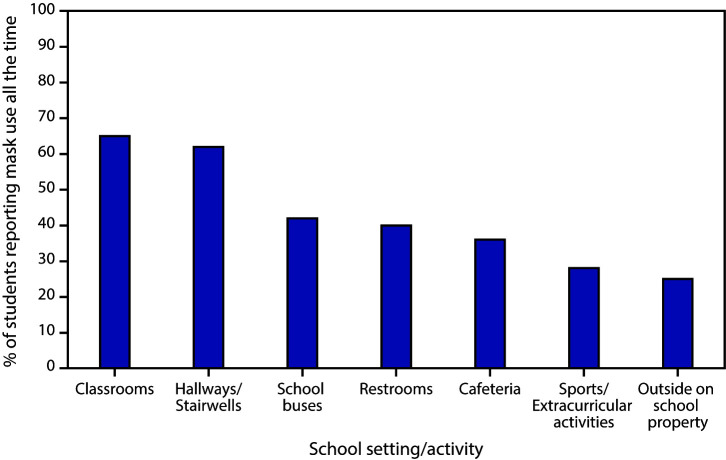
Mask wearing is a critical mitigation strategy in preventing the introduction and spread of SARS-CoV-2, the virus that causes coronavirus 2019 (COVID-19), within school settings. In October 2020, a sample of 3,953 middle and high school students aged 13–21 years who were attending in-person classes were asked about mask use by fellow students in several settings. Approximately 65% of students reported that fellow students wore a mask “all the time” in the classroom and in hallways or stairwells. However, reported use of masks all the time was lower in other indoor locations, including school buses (42%), restrooms (40%), and the cafeteria (when not eating) (36%). Reported observed mask use all the time was lowest during sports or extracurricular activities (28%) and outside on school property (25%).

**Source:** Falcon Goal 1 Student Web Panel Survey, October 1–24, 2020.

